# Use of *in vitro* derived human neuronal models to study host-parasite interactions of *Toxoplasma gondii* in neurons and neuropathogenesis of chronic toxoplasmosis

**DOI:** 10.3389/fcimb.2023.1129451

**Published:** 2023-03-08

**Authors:** Sandra K. Halonen

**Affiliations:** Department of Microbiology and Cell Biology, Montana State University, Bozeman, MT, United States

**Keywords:** human neurons, human iPSCs, spontaneous cystogenesis, bradyzoites, cerebral organoids

## Abstract

*Toxoplasma gondii* infects approximately one-third of the world’s population resulting in a chronic infection with the parasite located in cysts in neurons in the brain. In most immunocompetent hosts the chronic infection is asymptomatic, but several studies have found correlations between *Toxoplasma* seropositivity and neuropsychiatric disorders, including Schizophrenia, and some other neurological disorders. Host-parasite interactions of bradyzoites in cysts in neurons is not well understood due in part to the lack of suitable *in vitro* human neuronal models. The advent of stem cell technologies in which human neurons can be derived *in vitro* from human induced pluripotent stem cells (hiPSCs) or direct conversion of somatic cells generating induced neurons (iNs), affords the opportunity to develop *in vitro* human neuronal culture systems to advance the understanding of *T. gondii* in human neurons. Human neurons derived from hiPSCs or iNs, generate pure human neuron monolayers that express differentiated neuronal characteristics. hiPSCs also generate 3D neuronal models that better recapitulate the cytoarchitecture of the human brain. In this review, an overview of iPSC-derived neurons and iN protocols leading to 2D human neuron cultures and hiPSC-derived 3D cerebral organoids will be given. The potential applications of these 2D and 3D human neuronal models to address questions about host-parasite interactions of *T. gondii* in neurons and the parasite in the CNS, will be discussed. These human neuronal *in vitro* models hold the promise to advance the understanding of *T. gondii* in human neurons and to improve the understanding of neuropathogenesis of chronic toxoplasmosis.

## 1 Introduction

*Toxoplasma gondii* is an intracellular protozoan parasite with approximately one-third of the worlds’ population chronically infected. In chronically infected individuals, the parasite resides in the central nervous system (CNS) within neurons in cysts, that persist for the lifetime of the individual (Ferguson and Hutchison, 1987). In immunocompromised individuals, such as AIDS patients or individuals undergoing chemotherapy, the parasite can reactivate from the cyst stage and differentiate into the rapidly replicating tachyzoite stage causing a severe to potentially fatal encephalitis (Luft and Remington, 1992). The chronic infection in immunocompetent individuals is typically asymptomatic, though several studies have found correlations between *Toxoplasma* seropositivity and neuropsychiatric disorders such as Schizophrenia, prenatal depression, and suicidal thoughts and the chronic infection has also been associated with cryptogenic epilepsy and mild cognitive effects in elderly individuals, further indicating the chronic infection exerts effects on neuronal activity in the central nervous system (Yazar et al., 2003; Groer et al., 2011; Okusaga et al., 2011; Pedersen et al., 2012; Torrey et al., 2012; Beste et al., 2014; Sutterland et al., 2015; Xiao et al., 2018; Burgdorf et al., 2019; Oncu-Oner and Can, 2022). The mechanisms by which the parasite affects neuronal activity and CNS functions are not well understood.

Neurons are the predominant host cell for the bradyzoite stage and cysts in the chronic infection (Melzer et al., 2010; Cabral et al., 2016). Despite the central importance of bradyzoites and cysts in neurons in the chronic infection, these stages of the parasites’ life cycle, remain poorly understood. Studies on the effects of *T. gondii* infection in neurons is primarily derived from *in vivo* studies in mice. Early ultrastructural studies done in chronically infected mice found the synapse of infected neurons containing cysts remains intact, indicating parasite infection does not affect neuronal transmission (Sims et al., 1989). More recent *in vivo* studies in chronically infected mice however found neurons infected with the cyst stage are functionally silenced, have altered neurotransmitter levels and neural connectivity, and there is evidence of disruption of glutamate regulation by astrocytes and a loss of perisomatic inhibitory synapses, all indicating the parasite significantly alters neuronal functions (Prandovszky et al., 2011; Haroon et al., 2012; Brooks et al., 2015; David et al., 2016; Carrillo et al., 2020). Studies of *Toxoplasma-*infected neurons *in vitro* are limited to the study of the parasite in either primary neurons from rodents or human neuronal cell lines which do not display mature neuron characteristics (Sahm et al., 1997; Creuzet et al., 1998; Fagard et al., 1999; Schluter et al., 2001). Neither of these *in vitro* culture systems are adequate for study of effects of bradyzoites and cysts on human neuronal functions. A study in human neurons demonstrated neurons could support cyst development but was inadequate for long term culture needed for cyst maturation or the study of impact on neuronal functions (Halonen et al., 1996). Finally, the recent finding that bradyzoites and cysts are dynamic and heterogeneous entities in the chronic infection, as opposed to static structures as long thought, highlights the lack of understanding of these crucial stages and underscores the need for better *in vitro* neuronal models in which these stages of the parasites life cycle in human neurons can be studied (Watts et al., 2015).

A better understanding of the host-parasite interactions of *T. gondii* in human neurons and of the parasite effects on neuronal function is limited due to lack of suitable *in vitro* human neuronal models. The advent of cellular reprogramming technologies in which somatic cells can be used to derive functional human neurons *in vitro*, either *via* induced pluripotent stem cells (iPSCs) or *via* direct conversion of somatic cells to induced neurons (iNs), affords the opportunity to develop *in vitro* human neuronal culture systems to better understand host/parasite interactions of *T. gondii* in human neurons. Both hiPSC-derived neurons and iNs can generate two-dimensional (2D) neuronal monolayer cultures enabling cellular and molecular mechanistic studies to be done while hiPSC can also be used to derive three-dimensional (3D) cerebral organoids, affording disease modeling studies of neurological effects of *T. gondii* in the brain to be conducted. The use of stem cell technologies in Parkinson’s Disease (PD), Alzheimer’s Disease (AD), and Schizophrenia, has revolutionized the study and understanding of these neurological disorders (Brennand et al., 2011; Logan et al., 2019; Ferrari et al., 2020; Powell et al., 2020; D'Souza et al., 2021; Legault et al., 2022). These cellular reprogramming approaches have not been widely used in the study of *T. gondii* in the central nervous system (CNS) although they offer many of the same benefits such as providing an *in vitro* source of differentiated, mature, functional human neurons, enabling mechanistic studies, drug discovery and disease modeling studies, to be done.

Here, a review of iPSC-derived neuron and iN protocols leading to 2D neuronal monolayer cultures and hiPSC-derivation of 3D cerebral organoids models will be given with the goal of this review to summarize differentiation strategies of hiPSC-derived neurons, iNs, and 3D brain organoids, and to encourage the utilization of these *in vitro*-derived human neuronal models to address outstanding questions about host-parasite interactions of *T. gondii* in the human neuron host cell. A few *Toxoplasma* studies have used these approaches and a summary of the findings and potential applications of these 2D and 3D human neuronal models to address outstanding questions about the biology of *T. gondii* in neurons and neuropathogenesis of *T. gondii* chronic infection will be discussed. These cellular reprogramming approaches to generate human neuronal *in vitro* models hold the promise to advance the understanding of *T. gondii* interactions in human neurons and of neuropathogenesis of chronic toxoplasmosis.

## 2 Neurodifferentiation strategies

**Figure 1:**
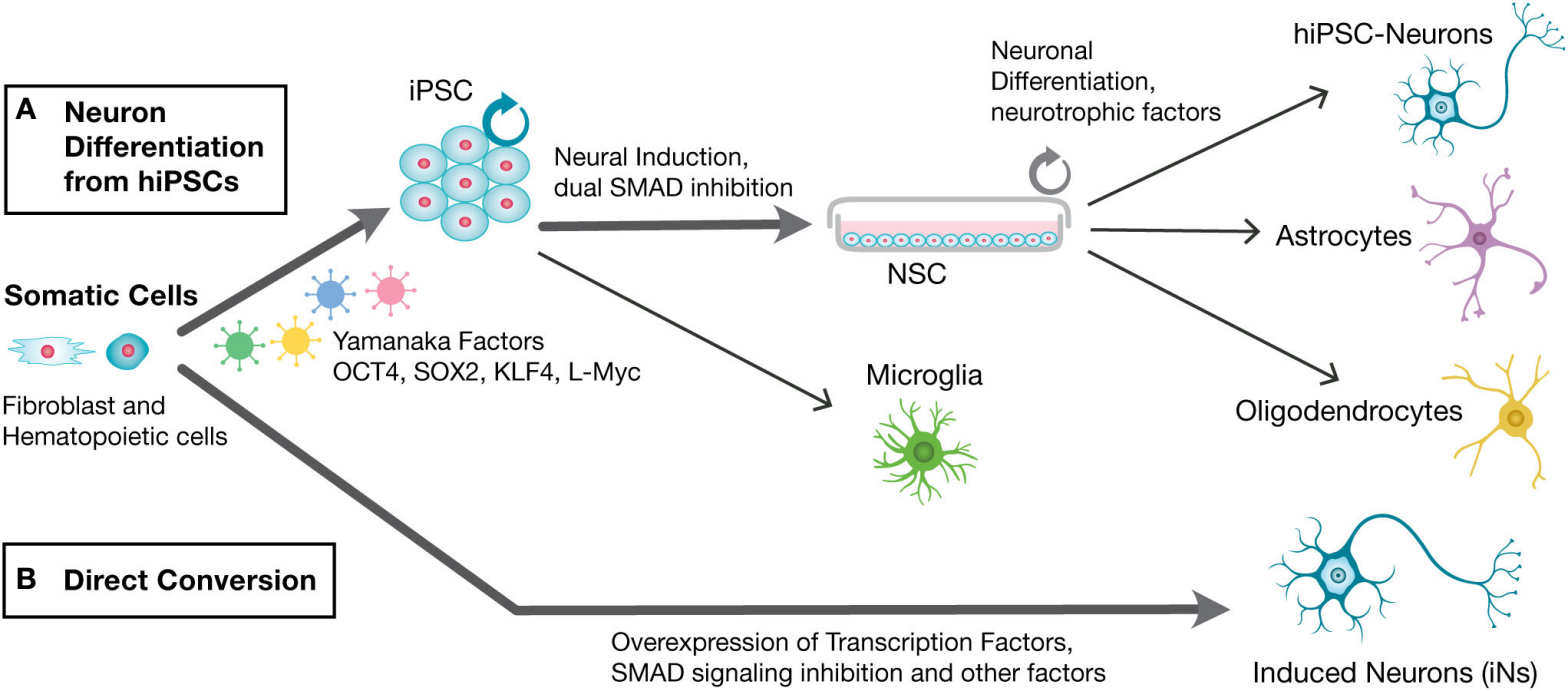
Schematic of steps involved in the generation of *in vitro* generation of human neurons from hiPSCs and direct conversion of induced neurons (iNs). Human primary fibroblasts or other somatic primary cells are used to generate human neurons *in vitro via* **(A)**. Neuronal differentiation from hiPSCs or **(B)**. Direct conversion into iNs. Neuronal differentiation from hiPSCs involves first reprogramming into hiPSCs *via* Yamanaka Factors, followed by neural induction into neural stem cells (NSCs) *via* dual SMAD inhibition, and differentiation with the aid of neurotrophic factors to generate hiPSC-neurons. hiPSCs-generated NSCs can also generate astrocytes and oligodendrocytes, while microglia can be generated from hiPSCs. Direct conversion into iNs involves overexpression of neuronal transcription factors, SMAD inhibition and other factors, to generate Induced Neurons (iNs).

Human neurons are generated *in vitro* either from human induced pluripotent stem cells (hiPSCs) or *via* direct conversion producing iNs (**Figure 1**). Both approaches generate 2D monolayers of relatively pure populations of differentiated, functional neurons. In addition, 3D organoid cultures can be produced from hiPSCs. Here an overview of methods used in the generation of hiPSC-derived neurons vs. direct conversion of iNs will be given, with a comparison of advantages and disadvantages of each approach.

### 2.1 hiPSC-derived neuronal cells

Human somatic cells exposed to Yamanaka factors, OCT4, KLKF4, SOX2 and c-MYC (OKSM), are reprogrammed into iPSCs which can be differentiated into different cell lineages, as first demonstrated in 2007 (Takahashi et al., 2007). In the years since the advent of iPSC technology refinements and improvements have occurred (Mertens et al., 2016; Traxler et al., 2019). Most differentiation protocols involve application of extrinsic factors to guide the differentiation process towards a cell fate, mimicking the regionalization processes that occur during the developmental process, followed by specific growth factors. In brief, hiPSC can be neutralized *via* dual SMAD signaling inhibition producing neural stem cell (NSCs), followed by addition of specific growth factors such as BDNF and GDNF and cAMP to induce differentiation into postmitotic neurons (**Figure 1A**) (Chambers et al., 2009; Shi et al., 2017). Differentiation into postmitotic neurons from NSCs typically takes 3-4 weeks. NSCs are multipotent and can be differentiated into astrocytes or oligodendrocytes with differentiation to these glial cell types typically requiring at least 60 days in *in vitro* culture (Hirbec et al., 2020). hiPSCs can be induced toward the mesodermal lineage and microglia generated, *via* specific growth factors, in about 15 days (Abud et al., 2017; Douvaras et al., 2017; Pandya et al., 2017).

### 2.2 Induced neurons

Human neurons can be generated *in vitro* through direct conversion of somatic cells into iNs (**Figure 1B**). Human fibroblasts can be converted into iNs *via* a combination of 3 transcription factors, BRN2, ACSL1, and MYTL1, the BAM reprogramming factors, in addition to the neurodifferentiation factor, NEUROD1 (Pang et al., 2011). It is now understood that during direct conversion, so called pioneering transcription factors bind closed chromatin structures and coordinate the binding of secondary transcription factors, to initiate a new cell fate (Mertens et al., 2016). Now, numerous conversion strategies have been developed involving the use of differing combinations of pioneering transcription factors, small molecules, and miRNAs (Schluter et al., 2001; Vierbuchen et al., 2010; Ladewig et al., 2012; Wapinski et al., 2013; Zhao et al., 2015). Most direct conversion protocols generate iNs from fibroblasts as the somatic cell, but diverse cells have been used for generating iNs, including blood cells, glial cells, pericytes and post-mortem brain tissues (Zhao et al., 2012; Rose et al., 2018; Traxler et al., 2019).

### 2.3 Generation of neuron subtypes from iNs and hiPSC-iNs

Subtype specific neurons, including glutaminergic, GABAergic, dopaminergic, and serotonergic neurons, can be generated either *via* from hiPSCs or iNs. Initial methods to derive neuronal subtypes from hiPSCs first created NSCs and then derived glutaminergic, GABAergic, and serotonergic neurons *via* mimicking developmental cues (Tao and Zhang, 2016). However, hiPSCs-NSC derived neuronal subtypes required long differentiation times leading to generation of heterogenous cell populations. More recently direct conversion of hiPSC to iNs (iPSC-iNs), called forward programming, has been found to generate highly pure populations of specific subtypes of neurons in an easy, reproducible manner (Flitsch et al., 2020; Canals et al., 2021). Forward programming is done *via* applying overexpression of lineage-specific transcription factors (TFs). This TF-based approach can generate dopaminergic neurons, glutaminergic neurons, GABAergic neurons and motor neurons from hiPSCs (Canals et al., 2021). Direct conversion of iNs similarly has been accomplished *via* the use of pioneering transcription factors in combination with factors shown to be important in neural subtype determination, with protocols to generate dopaminergic, GABAergic, and serotonergic neurons, now available (Vierbuchen et al., 2010; Caiazzo et al., 2011; Pfisterer et al., 2011; Victor et al., 2014; Mertens et al., 2015; Vadodaria et al., 2016; Xu et al., 2016; Mertens et al., 2018).

### 2.4 Cerebral organoids: 3D neuronal models

3D cerebral organoids contain multiple brain-specific cell types, obtain specialized function, and achieve an organ-like organization and overcome of the limitations of 2D neuronal monolayers (Liu et al., 2018; Logan et al., 2019; Pacitti et al., 2019). Cerebral organoids are derived from hiPSCs *via* both guided and unguided methods (Koo et al., 2019; Qian et al., 2019). Guided methods are dependent upon iPSCs intrinsic ability to assemble and differentiate towards the neuronal fate and yield cerebral organoids with the major features of the developing cortex, such as the outer subventricular zone and neural subtypes constituting all 6 cortical layers, formed (Koo et al., 2019; Sidhaye and Knoblich, 2021). In unguided methods, region-specific cerebral organoids are directed by region-specific growth factors, differentiation factors and specific cellular inhibitors. Protocols to generate region-specific cerebral organoids, including forebrain, midbrain, cerebellar, hippocampal, and hypothalamic organoids, are now established (Muguruma et al., 2015; Sakaguchi et al., 2015; Qian et al., 2016; Qian et al., 2018).

Cerebral organoids have a wider application for *in vitro* neurological disease modeling than 2D monolayers and have been successfully used in Alzheimer’s Disease, Parkinson’s Disease, and Schizophrenia (Choi et al., 2014; Tieng et al., 2014; Lee et al., 2016; Raja et al., 2016; Stachowiak et al., 2017; Amin and Pasca, 2018). Disadvantages of organoids include the need for more complex methods for characterization and analysis and the cost and the time to generate cerebral organoids.

### 2.5 hiPSC-neurons vs. iNs 2D neuronal models: Advantages and disadvantages

hiPSC-neurons and iNs are attractive *in vitro* 2D neuronal models for studying neuronal diseases because of their human origin, affording the ability to conduct mechanistic studies and screen for therapeutic targets, in human neurons. hiPSC-neurons and iNs have distinct advantages and disadvantages in terms of time to generate cells, capacity for expansion, number of cells that can be generated, epigenetic status and capability as models for neurological diseases (Mertens et al., 2016; Mertens et al., 2018; Traxler et al., 2019). The choice of the *in vitro* neuron culture method used, should be balanced with the research questions addressed and the practical experimental needs, to determine if these methods are worth the time and effort. Foremost amongst these considerations when thinking of using hiPSC or iN approach, are those of time and cost efficiency, expandability, epigenetic effects, and age-related phenotypes, each of which are briefly discussed below and summarized in **Table 1**.

**Table 1:** Comparison of iPSC-derived neurons vs. iNs.

Property	iPSCs or NSCs	iNs
**Cell Source**	Fibroblasts, keratinocytes, dental pulp cells, blood cells, renal epithelial cells	Fibroblasts, hepatocytes, adipocytes, pericytes, astrocytes and iPSCs
**Time to Generate neurons**	4-6 months from fibroblasts 3-6 weeks from iPSCs 1-4 weeks from NSCs	1-3 weeks plus maturation
**Capacity for expansion**	Infinite	Only at fibroblast stage as the derived neurons are postmitotic
**Potential numbers of cells**	Infinite	Limited by the expandability of fibroblasts and conversion efficiency
**Capacity to generate different neural subtypes**	Yes	Yes
**Diversity and mosaicism**	iPSC line is a single cell-derived clone	Reflects the cellular diversity of the donor tissue
**Epigenetic status**	Identity and age erased	Cell type identity erased; details of epigenome unknown
**Capacity to model neurodevelopment**	Yes	No
**Capacity to model ageing?**	No; cells are rejuvenated	Yes, ageing signatures preserved
**Capacity for 3D Cerebral Organoid?**	Yes, including forebrain, midbrain, cerebellar, hippocampal, and hypothalamic organoids	No

#### 2.5.1 Time and cost efficiency of hiPSCs vs. iNs

The generation of iPSC clones typically takes 2-3 months, but they can then be expanded indefinitely and provide an infinite supply of cells. Differentiation of iPSCs to neurons takes 6-15 weeks. NSCs can be established in about 2-3 weeks which can then be used as a stable intermediate cell line that can generate neurons in 3-4 weeks. Several iPSC and NSC cell lines are commercially available from ATCC (www.atcc.org), WiCell, (www.wicell.org), a variety of Schizophrenia-derived PSC cell lines are publicly available (Dobrindt et al., 2021) and several iPSC cell lines are available from NIH Center for Regenerative Medicine Program (https://commonfund.nih.gov/stemcells/lines). Conversely, iNs can be generated more quickly with direct conversion of fibroblasts to iNs generated in 1-3 weeks or with another 5-6 weeks required to generate fully mature neurons (Mertens et al., 2018; Traxler et al., 2019).

#### 2.5.2 Expandability and cell numbers

As iPSCs can be expanded infinitely and NSCs are self-renewing, both iPSC and NSCs can generate large numbers of neurons and thus suitable for metabolomic, proteomic, transcriptomic, and genomic analyses. Conversely, direct iN conversion does not involve an expandable, intermediate stage and given the neurons generated are post mitotic, the direct conversion of iNs approach is limited in the numbers of neurons that can be produced.

#### 2.5.3 Epigenetic and age-related effects

iPSC reprogramming involves chromatin remodeling that causes the epigenetic state of the cell to ‘reset’ into an embryonic-like state, with multiple rounds of cell division thought to select for and repair macromolecular damage, leaving iPSCs into what has been called a ‘rejuvenated cell’ (Mertens et al., 2018; Traxler et al., 2019). Conversely, iNs conserve the age-related epigenetic landscape and other cellular properties from the cell of origin and does not erase the cellular aging markers, with iNs derived from fibroblasts showing the transcriptomic and functional signatures of the age of the parent fibroblast (Mertens et al., 2015). The age-related epigenetic landscape of iNs may have advantages for disease modeling type studies where aged, patient-specific neurons in culture are desired.

## 3 hiPSC and iNs: new *in vitro* human neuronal models to study host/parasite relationship of *T. gondii* in neuron host cell and neuropathogenesis

These 2D and 3D *in vitro* human neuronal models open new possibilities for investigating *T. gondii* in human neurons and neuropathogenesis of *T. gondii* chronic infection. A few studies with *T. gondii* have been conducted using these *in vitro* human neuronal models which demonstrate their potential use in the study of *T. gondii* in neuronal cells. A brief description of each of these studies are given below, followed by a discussion of how these approaches could be applied to address outstanding questions of *T. gondii* in the brain, which is summarized in **Table 2**.

**Table 2:** Outstanding questions of *T. gondii* in neurons and in the CNS that could be addressed using 2D and 3D Human Neuronal Models.

	2D Monolayers	3D Organoids
**A. Bradyzoite and Cyst Biology**	1. Allows kinetic studies where temporal events that occur during bradyzoite differentiation and cystogenesis (1 day-14 days p.i.) can be studied 2. Allows longitudinal studies of bradyzoites in mature cysts to be studied to gain knowledge of cyst life histories 3. Amenable to live cell microscopy allowing dynamic events occurring within cysts such as bradyzoite motility and intraneuronal trafficking of bradyzoites to be discerned	1. Allows for mature cyst development 4 weeks facilitating study of mature cysts 2. Longitudinal studies of mature cysts (4 weeks) could be done although imaging would be challenging 3. Allow bradyzoite replication and patterns of cyst growth in mature cysts to be studied
**A. Host/parasite interactions in neurons**	1.Dissect neuronal host-parasite interaction of bradyzoites and cysts in neuron host cell 2. With iNs, can investigate effects of the parasite in neurons from individuals with neurological disorders	
**C. Neuropathogenesis**	1.Study effects of bradyzoites on neurotransmission and assessment of impacts on neuronal structures such as dendrites and synapses 2.Study effects of parasite in region-specific neurons such Dopaminergic, GABAergic, or Serotonergic neurons	1.Study effects of parasite infection on neurotransmission and neuronal networks 2. Investigate interactions between infected neurons and astrocytes
**D. Drug Discovery**	Screening and Evaluation of anti-bradyzoite and cyst drugs	Evaluate drugs effective against mature cysts

### 3.1 Neuronal monolayers: 2D human neuronal models

#### 3.1.1 Use of hiPSC-derived neurons

**Figure 2:**
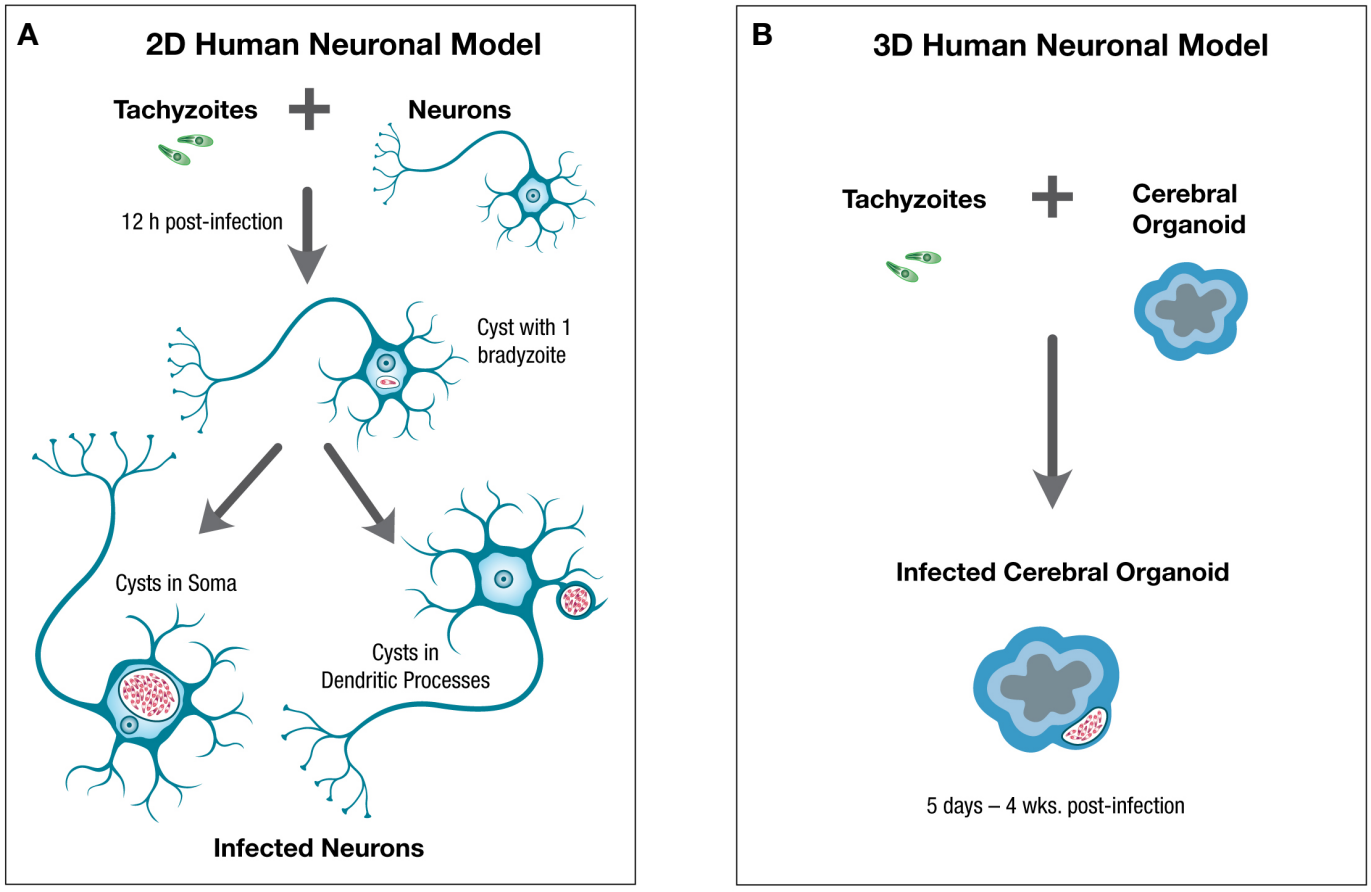
Schematic of 2D and 3D human neuronal *in vitro* models for the study of *T. gondii* in neurons and the central nervous system. Human neuronal *in vitro* models can be generated for the study of *T. gondii* in human neurons and the central nervous system using **(A)**. 2D human neuronal monolayers or **(B)**. 3D human neuronal models. For 2D neuronal models hiPSC-derived neurons can be infected with tachyzoites which convert to bradyzoites within 12hr post-infection (p.i.), leading to spontaneous cystogenesis with mature cysts located in the neuronal soma and dendritic processes by 4 days p.i. and which can be maintained for up to 14 days p.i. For 3D human neuronal models, cerebral organoids can be infected with tachyzoites which convert to bradyzoites and generate mature cysts by 5 days p.i. with cysts persisting in the outer layer of cerebral organoids, where mature neurons and some glial cells are found, for 4 weeks post-infection or longer. Tachyzoites are green, bradyzoites are red.

Human neurons were derived from NCRM-1 cells, a neural stem cell (NSC) line obtained from NIH, using a similar protocol as outlined in **Figure 1A** (Tanaka et al., 2016; Halonen, 2017). Human neurons were infected with *T. gondii* tachyzoites (type II strain) and at low parasite to host cell ratios (1:100), the majority of infected neurons supported spontaneous cystogenesis, with tachyzoite to bradyzoite stage conversion beginning within the first 12 hrs. post infection (p.i.) and mature cysts generated by 96h p.i. Mature cysts developed near the neuronal soma and in the dendritic processes and could be maintained in neurons for up to 14 days p.i. (**Figure 2A**).

This study demonstrated that hiPSC-derived human neurons is an effective *in vitro* model to study spontaneous cystogenesis and host/parasite interactions during cystogenesis and of mature cysts, in the human neuron host cell. As this method generates differentiated neurons generated from NSCs, a self-renewing population, this method has the advantage of expandability, capable of generating large numbers of human neurons and thus conducive to transcriptomic, proteomic, and other such analyses.

#### 3.1.2 Use of schizophrenia-derived iNs to study *T. gondii* in neurons

Peripheral fibroblasts were used to generate iNs from several patients with genetic variations linked to schizophrenia (SZ) and iNs from normal controls, and growth of *T. gondii*, RH strain, in disease vs. normal controls iNs, characterized (Passeri et al., 2016). Tachyzoites infected and generated cysts in both SZ-iNs and normal iNs, with some differences in growth found in the SZ-iNs vs. normal-iNs.

This iN study demonstrated mature human neurons can be generated from peripheral fibroblasts from patients with various brain disorders. The use of patient-derived iNs allows the heterogeneity of disease-associated phenotypes to be incorporated into investigation of molecular mechanisms underlying individual predisposition to *Toxoplasma* infection and various brain disorders.

### 3.2 Brain organoids: 3D human neuronal models

Two studies have investigated using 3D human neuronal models to study *T. gondii* infection in the brain (Seo et al., 2020; Correa Leite et al., 2021). In both 3D cerebral organoid models, infection with *Toxoplasma* tachyzoites, of both Me49 and RH strains, lead to bradyzoite differentiation and formation of mature cysts, with cysts persisting up to 4 weeks post-infection, within the cerebral organoids (**Figure 2B**).

Both studies demonstrated 3D organoid neuronal models can recapitulate the *in vivo* biology of *T. gondii* in the brain. Persistence of cysts for up to 4 weeks in these 3D models is a major advantage as these 3D models would allow studies on mature cysts to be conducted. Additionally, brain organoids contain multiple neural cell types, including neurons, neural progenitors, and glia, allowing dynamic interactions between neural cells to be studied and generation of neural cells with greater cell maturity and improved cell functionality (Liu et al., 2018; Pacitti et al., 2019). Infection of 3D organoid models also induced neural cell death, alteration in neural gene expression and triggering of the release of inflammatory markers in response to *Toxoplasma* infection, indicating these 3D neuronal organoids are also good models to address more complex interactions of the parasite in the brain.

### 3.3 Potential of 2D and 3D human neuronal models for *T. gondii* studies

#### 3.3.1 Use of 2D neuron monolayers to study *T. gondii* host/parasite interactions

2D neuronal models derived from hiPSCs offer many advantages to study *T. gondii* in neurons, such as the ability to create monolayers of relatively pure neurons that exhibit differentiated neuronal morphology that are conducive to single-cell or population-based assays and afford opportunities to address questions about the biology of bradyzoites and cysts in neurons. A major advantage of this *in vitro*-derived human neuron culture is to allow neuron host-cell specific responses to *Toxoplasma* infection to be addressed. Several recent studies indicate neuron-specific responses are important. For example, the protein effector export of the bradyzoites stage was found to be slightly different in neurons vs. fibroblasts with indication the nuclear effector stability or effector export was less efficient in neurons (Mayoral et al., 2020). Additionally, a novel mechanism of IFN- induced bradyzoite formation and cyst conversion, *via* depletion of intracellular glutamine, was recently identified in human glutamatergic neurons (Bando et al., 2021). As it is known the molecular mechanism of IFN-response is different in mice and humans these *in vitro-*derived human neurons would also facilitate other studies investigating mechanisms of IFN-effects on *T. gondii* in human neurons (Ohshima et al., 2014; Saeij and Frickel, 2017; Bando et al., 2018). Other advantages of *in vitro*-derived human neurons are they form monolayers that are amenable to microscopical analysis and would allow host/parasite interactions of bradyzoites in cysts in neurons during encystment and of mature cysts, to be addressed. Additionally, this *in vitro* human neuron culture model facilitates cyst development up through 2 weeks and would facilitate longitudinal studies of individual cysts in neurons in which dynamic aspects of bradyzoites and cysts could be studied, and could yield new information and insights into life histories of cysts in neurons. Other advantages of 2D human neuronal monolayers are summarized in **Table 2**.

#### 3.3.2 Use of 3D neuronal models to study *T. gondii* interactions in the CNS

Cerebral organoids would allow study of fully mature cysts as one of the limitations of the 2D human neuron monolayer for the study of *T. gondii* infection in neurons is the inability to maintain cysts for longer than 14 days post-infection, while 3D cerebral organoids allowed for cysts to persist for at least 4 weeks post-infection (**Figure 2B**). Thus, cerebral organoids may allow studies addressing behavior of bradyzoites in mature cysts, such as bradyzoite replication and patterns of cyst growth, to be studied. Cerebral organoids would also allow more complex questions of neuropathogenesis of *T. gondii* in the brain to be addressed such as the effects of infection on neuronal networks and the interactions between infected neurons and astrocytes. As neuron-astrocyte interactions have been shown to be involved in the impact of the parasite on neurotransmission, the ability to address the role of astrocytes on neuropathogenesis of *T. gondii* is an attractive aspect of 3D cerebral organoid models (David et al., 2016). Advantages of 3D cerebral organoids to address questions of *T. gondii* interactions in human CNS is summarized in **Table 2**.

#### 3.3.3 Limitations of 2D and 3D human neuronal models

While 2D and 3D neuronal models have the potential to create *in vitro* human neuronal models to address outstanding questions of host-parasite interactions of *T. gondii* in neurons and neuronal tissues, there are distinct limitations of each model, especially as applies to *T. gondii* studies, which need to be considered, as stipulated below.

##### 3.3.3.1 Conversion efficiency of 2D neuronal models

Low conversion efficiencies often occur with differentiation protocols for hiPSC-neurons and iNs. For hiPSC-human neurons, standardization of protocols and reagents have greatly improved protocol efficiencies and batch-to-batch consistency. Human iPSCs and NSCs for example are available from commercial sources such as ATCC and WiCell, with expansion and differentiation medias available and/or easy to make with the addition of defined supplements, making these protocols feasible for most *Toxoplasma* labs with established tissue culture. In evidence of this is the recent paper in which human iPSC-derived glutamatergic neurons were used to investigate molecular mechanisms of IFN- stimulated cyst formation in human neurons (Bando et al., 2021). iN protocols are not as well developed as hiPSCs, with iN protocols not standardized or reagents commercially available, and low conversion efficiencies and high variability in efficiency between reprogramming experiments, still a problem of most neural reprogramming protocols (D'Souza et al., 2021; Legault et al., 2022). Improved iN protocols are continually being developed and improved as for example, the recent induced dopaminergic neuron (iDANs) protocol which reports 90% conversion efficiency (Powell et al., 2021). These iDANs may provide a good *in vitro* model for midbrain dopaminergic neurons and addressing questions of association *T. gondii* and Schizophrenia. Thus, while iN protocols have improved in recent years, low conversion efficiencies are still common and need to be considered in context of the experimental question, if this method of generating *in vitro* human neurons is used.

##### 3.3.3.2 Issues of complexity of 3D organoids

As 3D cerebral organoids are generated from iPSCs they have many of the same limitations of hiPSC-derived neuronal 2D monolayers including differentiation efficiency and batch-to-batch variability. However, as with hiPSCs, in recent years 3D organoid protocols have progressed such that there are now simple brain organoid protocols and multiple specific brain-region protocols, that can be done in a reproducible and predictable manner (Sidhaye and Knoblich, 2021). Limitations however still exist. For one, 3D organoids have mixed populations of cells which complicate studies using bulk RNASeq, as cell identities of transcripts cannot be identified and thus these types of studies with 3D organoids would require more complex methods for analysis. However single-cell transcriptomics and a variety of other assays including live-cell imaging, electrophysiology, calcium dynamics and immunohistochemistry studies can be done, albeit they require more effort (Sloan et al., 2018).

##### 3.3.3.3 Lack of immune system components

Both 2D and 3D neuronal models lack an immune system component and thus do not fully recapitulate the *in vivo* environment of the CNS. Microglia, for example which play a crucial role in neuronal maturation and functioning and are implicated in neurodegeneration and psychiatric disorders such as Schizophrenia, are absent. Microglia can be generated from hiPSCs as diagrammed in **Figure 1A**, and some co-cultures of 3D hiPSC-organoids with hiPSC-derived microglia that infiltrate the organoid when added, have been used successfully in a few Alzheimer’s Disease studies (Abud et al., 2017; Song et al., 2019). As microglia have important roles in the biology of *T. gondii* in the brain, such as contributing to the loss of perisomatic inhibitory synapses following *T. gondii* infection (Carrillo et al., 2020), incorporation of microglia into 2D and 3D neurological models could be important for some studies. However, this would entail a complicated tissue culture process and the time and cost, needs to be carefully considered and balanced with the experimental question, in consideration of incorporation of microglia into these 2D and 3D human neuronal models.

## 4 Conclusions

The advent of stem cell technologies to generate human neuronal *in vitro* models has revolutionized the study of neurological disorders such as Parkinson’s Disease, Alzheimer’s Disease and Schizophrenia. The use of these human neuronal *in vitro* methods has a similar potential to advance the understanding of *T. gondii* in human neurons and of neuropathogenesis of the parasite in the CNS.

## Author contributions

SH wrote all sections of the manuscript, assembled information for the tables and designed the Figures with the aid of Kristen Drumheller, a Graphic Designer from University Communications Dept. at Montana State University.
